# Functional Near-Infrared Spectroscopy (fNIRS) in Objective Audiometry: A Scoping Review and Clinical Perspectives

**DOI:** 10.3390/audiolres16010003

**Published:** 2025-12-19

**Authors:** Tomáš Mimra, Martin Augustynek, Marek Penhaker, Lukáš Klein

**Affiliations:** 1 Department of Cybernetics and Biomedical Engineering, Faculty of Electrical Engineering and Computer Science, VSB—Technical University of Ostrava, 17. Listopadu 2172/15, 708 00 Ostrava, Czech Republicmarek.penhaker@vsb.cz (M.P.); lukas.klein@vsb.cz (L.K.); 2 ENET Centre—CEET, Faculty of Electrical Engineering and Computer Science, VSB—Technical University of Ostrava, 708 00 Ostrava, Czech Republic

**Keywords:** fNIRS, objective audiometry, BERA, scoping review, hearing assessment, pediatric audiology, cochlear implants, neuroimaging

## Abstract

Background: The objective assessment of hearing in non-cooperative populations, such as neonates, remains a challenge. While Brainstem Evoked Response Audiometry (BERA) is the gold standard, its sensitivity to motion artifacts necessitates alternatives. Objective: This scoping review maps the current literature on functional near-infrared spectroscopy (fNIRS) as a supplementary method in objective audiometry. Data Synthesis: fNIRS shows potential to detect cortical hemodynamic responses, particularly to complex stimuli like speech, which BERA cannot fully assess. Key advantages include motion tolerance and suitability for pediatric and cochlear implant populations. However, the literature reveals significant heterogeneity in stimulation protocols and data processing. Evidence suggests fNIRS is better suited for assessing higher-level auditory processing rather than replacing BERA for threshold estimation. Conclusions: fNIRS is a promising complementary tool. However, due to the lack of standardized protocols and large-scale validation studies, it is not yet a direct clinical replacement for BERA. Future work must focus on protocol standardization and establishing robust normative data.

## 1. Introduction

Modern clinical audiology faces the persistent challenge of objectively and reliably assessing auditory function. This need is particularly urgent in populations whose ability to cooperate actively is limited, such as neonates, young children, or patients with cognitive or neurological disorders. For decades, the gold standard in this field has been Brainstem Evoked Response Audiometry (BERA), a method based on Electroencephalography (EEG) recording [1]. Despite its established position, BERA suffers from a number of well-documented limitations that significantly reduce its clinical utility and drives the search for more robust alternatives [2].

Among the most significant drawbacks of BERA are its high time consumption (often 30–60 min) [3], a considerable degree of subjectivity in the visual interpretation of results [4,5], and, above all, extreme sensitivity to motion and muscle artifacts. This problem is so fundamental that in uncooperative patients, especially children, it often necessitates performing the examination under sedation or general anesthesia. However, this procedure inevitably carries anesthesiological risks, increases the invasiveness of the procedure, and presents an additional logistical and financial burden [6,7]. These limitations define a clear research gap: the need for an objective, less artifact-sensitive, and more patient-friendly method.

In this context, Functional Near-Infrared Spectroscopy (fNIRS) is an exceptionally promising technology. fNIRS is a non-invasive optical neuroimaging technique that measures changes in the concentration of hemoglobin in the cerebral cortex associated with neural activity. Owing to its non-invasiveness, high tolerance to motion artifacts, and silent operation, fNIRS offers the potential to overcome many of the limitations of BERA [8,9].

While BERA assesses neural integrity at the brainstem level, fNIRS reflects cortical hemodynamic processing. Therefore, this review does not aim to present fNIRS as a direct competitor to BERA in terms of latency precision or threshold estimation, but rather to evaluate its capacity to provide supplementary functional information where BERA is limited.

Although several reviews have addressed fNIRS in specific sub-domains (e.g., solely in cochlear implants or autism), this paper aims to synthesize findings across these fields to provide a holistic view of its potential clinical integration as a complement to standard electrophysiological methods.

The aim of this article is to map and analyze the current state of knowledge on the use of fNIRS in objective audiometry. The paper reviews the theoretical foundations, analyzes key findings from recent studies, and critically discusses the clinical potential, limitations, and future directions.

## 2. Methodology

This study was conducted as a scoping review to map the available evidence on fNIRS in audiometry. The methodology followed the general principles of the PRISMA 2020 statement [10] for the search and selection process. A comprehensive and systematic search of the PubMed, Scopus, Web of Science, and IEEE Xplore electronic databases was performed in August 2025 to identify relevant studies published between January 2010 and August 2025. The search strategy employed a combination of keywords and Medical Subject Headings (MeSH) terms, where applicable, linked with Boolean operators (AND/OR). The specific search query included the following: ((“functional near-infrared spectroscopy” OR “fNIRS”) AND (“auditory” OR “hearing” OR “audiometry” OR “cochlear implant” OR “hearing aid” OR “auditory evoked potentials” OR “BERA”)).

**Figure 1:**
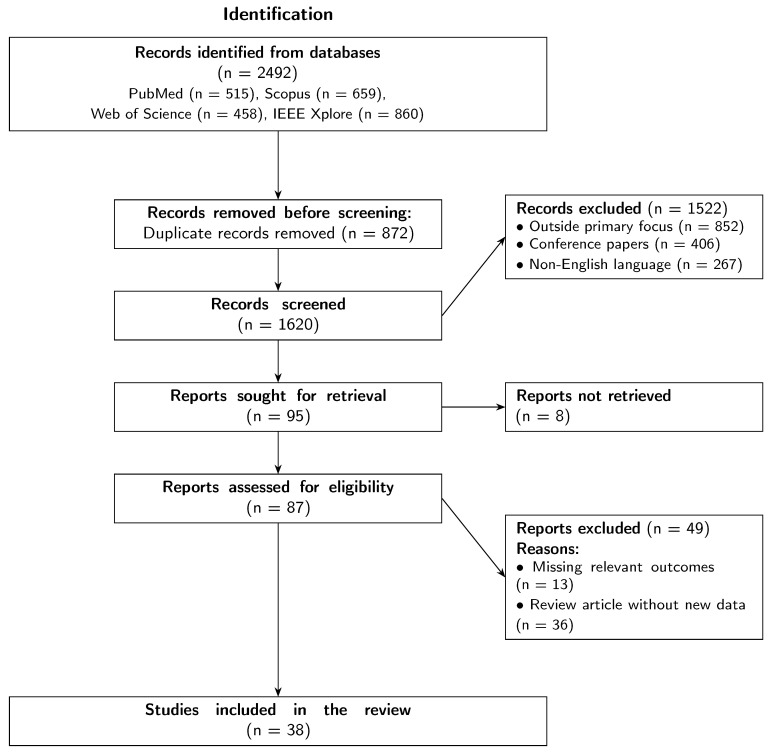
PRISMA 2020 flow diagram illustrating the process of identification, screening, eligibility assessment, and final inclusion of studies in the review.

The initial database search yielded 2492 records, as detailed in the PRISMA flow diagram (Figure 1). The breakdown of these records by database was as follows: PubMed (*n* = 515), Scopus (*n* = 659), Web of Science (*n* = 458), and IEEE Xplore (*n* = 860). Following the identification phase, all records were imported into a reference management software, and 872 duplicate records were removed. The remaining 1620 unique records underwent a title and abstract screening process conducted independently by two reviewers. The inclusion criteria for this stage were as follows: original research articles, review articles, or meta-analyses published in English that investigated the use of fNIRS for assessing auditory function in human subjects.

Relevant outcomes were defined as follows: (1) detection of significant hemodynamic changes (HbO/HbR) in response to auditory stimuli; (2) correlation of fNIRS responses with behavioral hearing thresholds; (3) identification of biomarkers for cortical plasticity (e.g., lateralization changes).

As shown in Figure 1, a total of 1522 records were excluded during this screening phase because they were outside the primary focus of the review (*n* = 852), were conference papers without an available full text (*n* = 406), or were not published in English (*n* = 267).

Following the initial screening, 95 reports were sought for full-text retrieval to determine eligibility. Of these, 8 reports could not be retrieved, leaving 87 full-text articles for detailed eligibility assessment, as illustrated in the “Eligibility” phase of Figure 1. The same two reviewers independently assessed these articles against the predefined inclusion criteria. Any disagreements between the reviewers at any stage of the selection process were resolved through discussion and consensus, with a third reviewer available for arbitration if necessary. During the full-text assessment, 49 reports were excluded. The primary reasons for exclusion were a lack of relevant outcomes (*n* = 13) and articles being review papers that did not contribute new data pertinent to this synthesis (*n* = 36). This rigorous selection process resulted in a final cohort of 38 studies that were included in the review. The entire workflow, from initial identification to final inclusion, is visually represented in Figure 1.

## 3. Theoretical Foundations of Imaging Methods

To understand the clinical potential of fNIRS in audiometry, it is essential to distinguish between the physiological mechanisms targeted by traditional methods and optical neuroimaging. This section outlines the fundamental operating principles of both electrophysiological and hemodynamic assessments, highlighting how their distinct temporal and spatial characteristics contribute to a more comprehensive evaluation of the auditory pathway.

### Complementarity of BERA and fNIRS

Auditory Evoked Potentials (AEP) and BERA provide direct insight into the functional state of the auditory pathway, focusing on early responses (within 10 ms) generated in the brainstem. The result is a characteristic waveform reflecting neural conduction integrity [11,12]. In contrast, fNIRS operates on the principle of neurovascular coupling, measuring hemodynamic changes (HbO/HbR) in the cortex that peak 6–8 s after stimulation [13,14].

The fundamental difference lies in their resolution and scope. BERA offers precise temporal resolution (milliseconds) and vertical localization (brainstem nuclei), whereas fNIRS provides superior spatial resolution of cortical topography. Thus, BERA answers “WHEN” and “IF” the signal reaches the brain, while fNIRS answers “WHERE” and “HOW MUCH” the cortex processes the signal [15]. Their combination provides a comprehensive view of brain function [16]. Table 1 summarizes these differences.

## 4. Current State of Research: Key Areas and Findings

The literature analysis reveals several key areas where fNIRS demonstrates its potential in audiological research and clinical applications. A summary of studies included in this review is provided in Table 2 and Table 3. It should be noted that these tables exclusively list the original research articles that met the inclusion criteria for data synthesis (*n* = 38). References cited solely for theoretical background, methodological guidelines, or clinical context are excluded from this summary to strictly focus on the analyzed empirical evidence.

### 4.1. Demographic Distribution: Adults vs. Pediatric Population

The primary parameter of the analysis is the distribution of studies between adult and pediatric populations. This ratio serves as a critical indicator of the clinical maturity of the method. While adult populations often serve for the validation of principles and normative studies, the pediatric population represents the target demographic for which fNIRS is primarily being developed, largely due to the potential to avoid sedation (Table 4).

Trend Analysis: The data indicates a significant prevalence of studies involving the adult population. A depth analysis of citations reveals that the “Adults” category also includes a specific subgroup of geriatric patients (e.g., [12,17]), where the effects of aging on central auditory processing (Central Presbycusis) are investigated. Pediatric studies are specifically concentrated in two areas: hearing screening in neonates and developmental disorders in older children. This disproportion suggests that while clinical demand is oriented towards children, methodological development is still primarily conducted on adult volunteers.

### 4.2. Experimental Paradigm: Block Design vs. Event-Related Design

The choice of experimental design is critical in fNIRS due to the slow hemodynamic response (peaking 6–8 s post-stimulus). The analysis of methodologies reveals a clear preference.

Trend Analysis: In contrast to EEG/BERA, where the Event-Related design dominates (to capture ERP waves such as N1, P2), fNIRS shows a clear predominance of Block Design. Citations such as [1,18] illustrate this contrast. While Martin describes “Speech-Evoked Potentials” (Event-Related), fNIRS studies like Bell et al. [19] utilize blocks of continuous speech. This dominance is a direct consequence of the physiology of neurovascular coupling—the metabolic change is too slow to effectively track rapid sequences of short stimuli without signal saturation (Table 5).

### 4.3. Types of Acoustic Stimuli: Speech vs. Tones vs. Noise

Citation analysis reveals that fNIRS is shifting from simple tone detection to more ecologically valid stimuli (Table 6).

Trend Analysis: The high volume of studies utilizing speech (natural, reversed, or in noise) supports the hypothesis that fNIRS is more suitable for assessing “higher” auditory functions. Studies by Bálint [20] and Mai [17] explicitly utilize “Speech-in-Noise” combinations, which are considered the most sensitive tests in modern audiology for detecting Hidden Hearing Loss and evaluating hearing aid benefits.

### 4.4. Compensatory Aids: Cochlear Implants (CIs) vs. Hearing Aids (HAs)

This represents one of the most significant categories where fNIRS demonstrates superiority over EEG (Table 7).

Trend Analysis: The data unequivocally indicates that fNIRS is the “technology of choice” for the Cochlear Implant (CI) population. Citations by Chen [21] and Basura [22] are pivotal in this regard. They demonstrate the ability to monitor cortical neuroplasticity with the implant activated, which is practically impossible with other methods (fMRI is often contraindicated or requires magnet explantation; EEG is artifact-ridden).

### 4.5. Specific Pathologies: Autism and Developmental Disorders

The final category concerns applications in neurodevelopmental disorders, where patient cooperation is limited (Table 8).

Trend Analysis: Although the absolute number of studies is lower, their clinical impact is high. The study by Lai et al. [23] identifies biomarkers (atypical lateralization) that could serve for early diagnosis. The low number of studies reflects the extreme difficulty of recruitment and testing in this specific population, rather than a lack of potential for the method.

### 4.6. Influence of Stimulation Protocols and Stimulus Types

One of the most crucial findings is that the successful detection of a response using fNIRS is critically dependent on the type and duration of the stimulus. The majority of studies indicate that while short, rapid stimuli like the “clicks” used in BERA are insufficient to elicit a robust hemodynamic response, more complex and longer-lasting stimuli lead to significant and well-detectable results. The hemodynamic response appears to be more sensitive to the complexity and semantic content of the sound. For instance, stimuli such as continuous speech, music, or complex tone sequences reliably evoke detectable hemodynamic changes in the auditory cortex and related association areas [18].

Research further indicates differences in cortical activation depending on stimulus specifics:

- Speech vs. Non-speech Stimuli: Comparing responses to speech (e.g., sentences, words) and acoustically similar but unintelligible sounds (e.g., reversed speech) allows for the isolation of areas specifically involved in language processing. Typically, stronger activation is observed in the left temporal lobe for intelligible speech.
- Frequency and Intensity Dependence: A subset of studies examining responses to pure tones of different frequencies and intensities helps map the tonotopic organization of the auditory cortex and objectively estimate the hearing threshold, albeit with less precision than BERA for rapid changes.
- Listening in Noise: Paradigms where a target stimulus (e.g., speech) is presented against a background of noise are particularly valuable. They allow measurement not only of auditory cortex activation but also the engagement of prefrontal areas, reflecting listening effort and cognitive load.

These findings suggest that fNIRS is better suited for evaluating higher cortical processes associated with sound perception and comprehension than for testing the basic integrity of the auditory pathway.

### 4.7. Applications in Pediatric Audiology

The strengths of fNIRS are particularly apparent in pediatric applications. Its non-invasiveness and motion tolerance permit the examination of young children without the need for sedation. For example, studies in children with autism spectrum disorders using fNIRS have revealed atypical hemispheric lateralization during speech processing [23]. Innovative approaches even combine fNIRS with virtual acoustic environments to investigate speech understanding in noise in children with hearing aids, offering new insights into neural mechanisms in simulated real-world conditions [19]. Further research utilizes fNIRS for the objective assessment of hearing abilities in newborns and infants, where traditional methods fail or are difficult to perform [24,25].

### 4.8. Utilization in Cochlear Implant and Hearing Aid Users

fNIRS is becoming a valuable tool for the objective evaluation of the benefits of hearing aids and for investigating central auditory plasticity following hearing loss, both in cochlear implant users and in patients with tinnitus [22]. It allows, for example, for the tracking of cortical neuroplasticity. A study in older adults, most with mild-to-moderate hearing loss, used fNIRS to assess the neuroplasticity of speech-in-noise processing after targeted training. The results showed that neural changes (e.g., reduced response in the left auditory cortex and increased functional connectivity) appeared even before significant behavioral improvement, demonstrating the method’s sensitivity for detecting neuroplastic changes [17].

In the context of cochlear implants (CIs), studies show that patterns of cross-modal reorganization (the engagement of the auditory cortex in processing other senses, e.g., vision, before implantation) can serve as predictors of clinical outcomes after CI placement [21].

Furthermore, fNIRS detects compensatory mechanisms and increased cognitive load during listening in challenging conditions, which helps to optimize implant settings [20,26,27].

Recent literature underscores the critical role of fNIRS in monitoring auditory cortical development. A 2025 review on pediatric audiology highlights that fNIRS is particularly effective in tracking cortical plasticity in children with cochlear implants, identifying biomarkers of cross-modal reorganization that correlate with speech perception outcomes [28]. These findings suggest that fNIRS can serve as a predictor of implantation success by monitoring how the auditory cortex adapts to electrical stimulation over time.

### 4.9. Multimodal Approaches (fNIRS-EEG)

A growing number of studies are using simultaneous EEG and fNIRS recordings to obtain a more comprehensive picture of auditory processing [16,29]. This approach allows for the correlation of fast electrical responses (e.g., the N1 wave) with slow hemodynamic changes, thus providing a better understanding of the relationship between neural activity and its metabolic impact [18,30]. Multimodal imaging thereby contributes to the identification of more robust biomarkers for neurological and psychiatric disorders [31].

## 5. Challenges, Limitations, and Future Directions

While the reviewed literature demonstrates the capacity of fNIRS to detect cortical responses to sound, several technical and methodological barriers impede its widespread transition from the research laboratory to routine clinical practice. The following subsections critically examine the quality of the current evidence, identify systemic issues regarding standardization, and outline the necessary steps for future technological development.

### 5.1. Quality of Evidence and Study Heterogeneity

While the reviewed studies highlight the potential of fNIRS, it is crucial to interpret these findings with caution. A significant limitation of the current body of literature is the high heterogeneity in study designs, particularly regarding stimulation protocols (block vs. event-related), probe placement, and signal processing pipelines. Many of the included studies report on relatively small sample sizes (*n* < 20) or lack robust control groups, which may inflate the perceived reliability of fNIRS findings. Furthermore, the absence of standardized reporting for signal-to-noise ratios and artifact rejection methods makes it difficult to assess the true effect sizes across studies.

Unlike previous reviews that focused on isolated clinical populations, this synthesis reveals that the lack of standardized protocols is a systemic issue affecting the entire field of objective audiometry, not limited to specific subgroups. Consequently, statements regarding the “reliability” of fNIRS should be understood in the context of research settings rather than established clinical utility.

### 5.2. Standardization and Robustness

A major obstacle is the lack of standardized stimulation protocols and analytical procedures. This includes the need for consensus on optimal stimulus parameters (e.g., duration, intensity, inter-stimulus interval), experimental designs (e.g., block versus event-related), and data processing pipelines, particularly for motion artifact correction and statistical analysis [32,33]. To ensure comparability of results between laboratories, a consensus on optimal measurement and data processing parameters is necessary. The fNIRS signal is also susceptible to contamination by motion and physiological artifacts (heartbeat, respiration). Their removal requires advanced signal processing methods, which are crucial for obtaining reliable data. Commonly used techniques include the following:

- Frequency Filtering: Application of a band-pass filter (typically 0.01–0.1 Hz) to remove slow drifts and high-frequency noise, including physiological signals such as heart rate (>0.8 Hz) and respiration (∼0.25 Hz).
- Short-Channel Signal Regression: Modern systems use optodes with a short separation (<1 cm) that primarily measure the signal from superficial tissues (scalp). This signal is subsequently used to “clean” the signal from long channels that measure brain activity, thereby effectively removing systemic physiological interference.
- Blind Source Separation Methods: Advanced algorithms like Independent Component Analysis (ICA) can decompose the signal into individual components, allowing for the identification and removal of those corresponding to artifacts [8,34].

Lack of Direct Comparative Studies: A significant gap in the current literature is the scarcity of studies directly comparing BERA and fNIRS sensitivity within the same patient cohort during a single diagnostic session. Most existing research focuses on validating fNIRS against behavioral thresholds or in isolation, rather than correlating it specifically with brainstem responses. Future research should aim to bridge this gap to establish clinical concordance between these two modalities.

Review Limitations: It should be noted that a significant number of potential studies (*n* = 264) were excluded due to language barriers (non-English). While this is common in systematic reviews, it may exclude valuable regional data or alternative protocols developed in non-English speaking countries. Future reviews should consider using advanced translation tools to include this “gray literature” for a more global perspective.

In addition to technical and methodological challenges, practical and economic barriers also hinder the wider clinical adoption of fNIRS. The acquisition costs for high-quality fNIRS systems, especially those with high-density optode arrays, are still relatively high and can exceed the price of standard EEG equipment [35]. A key obstacle is also the lack of reimbursement from health insurance providers, as the method is still perceived as experimental in most countries and lacks established billing codes [36]. Lastly, although the measurement itself is comfortable for the patient, the interpretation of complex hemodynamic data requires specialized personnel and time-consuming analysis, placing increased demands on resources and staff training [37].

### 5.3. Future Directions

Future research will focus on several key areas. Firstly, on the development and validation of robust, ideally automated, algorithms for real-time artifact correction [9,38]. Secondly, on the use of machine learning and artificial intelligence methods for the objective classification of responses, which would reduce subjectivity and accelerate the diagnostic process [39,40,41]. Thirdly, on conducting large-scale clinical validation studies to confirm the diagnostic accuracy of fNIRS in large patient cohorts. Furthermore, other promising directions are emerging:

- Wearable fNIRS Technology: The miniaturization of systems allows for the development of lightweight, portable devices that could monitor auditory processing in the patient’s natural environment (e.g., at home, at school), which would significantly increase the ecological validity of the measurements. However, these wearable systems also introduce challenges related to lower signal-to-noise ratios and the need for more advanced, robust algorithms to handle motion artifacts in unconstrained environments.
- Integration with Other Modalities: Combining fNIRS with other sensors, such as eye-tracking, can provide a more comprehensive view of the cognitive processes associated with listening, such as attention and listening effort.

These innovations promise to move fNIRS from the research laboratory closer to standard clinical practice.

## 6. Conclusions

This scoping review highlights that functional near-infrared spectroscopy and electroencephalography are not competing but rather complementary methods. While EEG/BERA remains an indispensable tool for the temporally precise assessment of auditory pathway integrity at the brainstem level, fNIRS is emerging as a powerful and viable technique for the objective evaluation of cortical sound processing.

A pivotal finding is that when using appropriately designed, complex, and sufficiently long acoustic stimuli, fNIRS can reliably and robustly capture cortical hemodynamic activity. Its key advantages—non-invasiveness, tolerance to motion, and silent operation—make it eminently suitable for clinical use in situations where traditional methods like BERA fail or are difficult to perform. This is particularly true for pediatric audiology, diagnostics in patients with neurodevelopmental disorders, and the objective evaluation of the benefits of hearing aids.

Work in the field is moving towards further standardization, the development of advanced analytical tools, and clinical validation. fNIRS has the potential to become a standard part of the diagnostic arsenal and to contribute to the development of personalized and more precise medicine, not only in audiology but also in related fields such as neurology and developmental psychology.

## Figures and Tables

**Table 1:** Comparison of EEG and fNIRS methods for measuring auditory evoked potentials.

Criterion	EEG/BERA	fNIRS
Temporal Resolution	Very high (milliseconds)	Low (seconds)
Spatial Resolution	High for brainstem nuclei; Low for cortical topography	Moderate to High for cortical surface (topography)
Artifact Sensitivity	High (motion, muscle)	Moderate (less sensitive to muscle)
Portability	Limited	High (compact systems)
Patient Comfort	Lower (gel, preparation)	Higher (dry optodes)
Clinical Standardization	High	Low (developing)

**Table 2:** Summary of representative studies included in the review (Part 1 of 2).

Ref.	Author (Year)	Article Title	Context and Significance
[1]	Martin, B.A. et al. (2008)	Speech-evoked potentials: from the laboratory to the clinic	Comprehensive review of speech-evoked potentials (specifically the P1-N1-P2 complex). It establishes the electrophysiological baseline for assessing central auditory processing of complex stimuli, serving as a key comparison for hemodynamic responses measured by fNIRS.
[2]	Swami, H. & Kumar, S. (2019)	Comparison of frequency-specific hearing thresholds between pure-tone audiometry and auditory steady-state response	Comparative study evaluating ASSR accuracy against behavioral audiometry, highlighting the need for reliable objective frequency-specific thresholds.
[3]	Chawda, U. et al. (2023)	A comparative study of OAE and BERA/ASSR as a screening of hearing loss among the children (<12 years of age)	Compares objective screening tools (OAE, BERA, ASSR) in pediatrics, underscoring limitations of current gold standards regarding time and testing conditions.
[4]	Karmacharya, S. et al. (2023)	Comparative study of Automated Auditory Brainstem Response (AABR) and BERA for Hearing Loss Detection in High Risk Infants	Focuses on automated vs. manual BERA reliability in high-risk infants, reinforcing the need for objective tools in non-cooperative populations.
[5]	Ramachandran, V. et al. (2011)	Communication Outcomes in Audiologic Reporting	Discusses subjectivity in interpreting audiologic data, supporting the argument that traditional methods suffer from observer bias.
[8]	Klein, F. (2024)	Optimizing spatial specificity and signal quality in fNIRS: an overview of potential challenges	Technical overview of fNIRS limitations (spatial specificity), framing it as a complementary tool (“where”) rather than a direct replacement (“when”).
[9]	Jie, Z. et al. (2020)	fNIRS Signal Motion Correction Algorithm Based on Mathematical Morphology and Median Filter	Proposes algorithms to clean fNIRS signals from motion artifacts, addressing a crucial technical challenge for awake pediatric testing.
[11]	Bigras, C. et al. (2022)	The electrophysiological markers of hyperacusis: a scoping review	Discusses electrophysiological markers to contrast physiological mechanisms between electrical (EEG) and hemodynamic imaging.
[12]	Jerger, J. & Lew, H.L. (2004)	Principles and clinical applications of auditory evoked potentials in the geriatric population	Establishes traditional AEP waveforms, providing a baseline for comparing the slower hemodynamic responses measured by fNIRS.
[13]	Kim, H.Y. et al. (2017)	Application of Functional Near-Infrared Spectroscopy to the Study of Brain Function	Explains the principle of neurovascular coupling and the 6–8 s delay, differentiating fNIRS temporal resolution from EEG.
[14]	Moreno-Castillo, M. et al. (2020)	The Hemodynamic Mass Action of a Central Pattern Generator	Elucidates hemodynamic principles, supporting the theoretical distinction between optical and electrical measurements.
[15]	Qin, Y. et al. (2024)	Optimizing spatial accuracy in electroencephalography reconstruction through diffuse optical tomography priors	Describes the synergy between EEG and optical methods, highlighting how fNIRS provides spatial location to complement EEG’s temporal precision.
[16]	Yeom, S.K. et al. (2017)	Spatio-temporal dynamics of multimodal EEG-fNIRS signals in the loss and recovery of consciousness	Demonstrates the power of combining EEG and fNIRS to track brain states, supporting the trend towards multimodal approaches.
[17]	Mai, G. et al. (2024)	Neuroplasticity of Speech-in-Noise Processing in Older Adults Assessed by fNIRS	Validates sensitivity to neuroplasticity; neural changes were detectable via fNIRS before behavioral improvements occurred.
[18]	Muñoz, V. et al. (2025)	Sound intensity-dependent cortical activation: implications of the electrical and vascular activity	Experimental validation that hemodynamic response amplitude scales with sound intensity, supporting objective threshold estimation.
[19]	Bell, L. et al. (2020)	fNIRS Assessment of Speech Comprehension in Children in Virtual Acoustic Environments	Demonstrates ecological validity by investigating speech comprehension in “virtual acoustic” noise, a task BERA cannot assess.
[20]	Bálint, A. et al. (2024)	Neural Correlates of Speech Comprehension in Normal Hearing Individuals and Cochlear Implant Users	Shows CI users exhibit higher cognitive load (frontal activation) in noise, helping optimize settings based on brain effort.

**Table 3:** Summary of representative studies included in the review (Part 2 of 2).

Ref.	Author (Year)	Article Title	Context and Significance
[21]	Chen, L. et al. (2016)	Cross-modal functional reorganization of visual and auditory cortex in adult cochlear implant users	Highlights fNIRS predictive value; shows visual cortex taking over auditory areas correlates negatively with CI outcomes.
[22]	Basura, G.J. et al. (2018)	Human central auditory plasticity: A review of fNIRS to measure cochlear implant performance	Synthesizes findings on how fNIRS tracks cortical changes in CI and tinnitus patients, supporting monitoring of long-term adaptation.
[23]	Lai, B. et al. (2024)	Atypical brain lateralization for speech processing at the sublexical level in autistic children revealed by fNIRS	Key clinical study showing children with ASD process speech with atypical hemispheric lateralization compared to neurotypical peers.
[24]	Oberman, L.M. et al. (2024)	Design and methodology for … Theta burst stimulation for auditory processing in adolescents with ASD	Discusses using neuroimaging to guide therapeutic interventions (stimulation) in adolescents with ASD.
[25]	Hirano, Y. & Tamura, S. (2021)	Recent findings on neurofeedback training for auditory hallucinations in schizophrenia	Reviews neurofeedback via fNIRS to treat auditory hallucinations, showing the method’s versatility in psychiatric audiology.
[26]	Cao, L. et al. (2025)	A dual-modality study on the neural features of cochlear implant simulated tone and consonant perception	Uses dual-modality imaging to provide insights into compensatory mechanisms for speech perception with degraded inputs.
[27]	Silva, L.A.F. et al. (2013)	Long latency auditory evoked potentials in children with cochlear implants: systematic review	Systematic review of electrical potentials in CI children, used to compare cortical reorganization findings with hemodynamic studies.
[28]	Lucarini, G. et al. (2025)	Imaging the developing brain with near-infrared spectroscopy in cochlear implanted children	Underscores the ability of fNIRS to image the developing brain and track cortical maturation for early intervention.
[29]	Kumar, C. et al. (2023)	Context-aware Multimodal Auditory BCI Classification through Graph Neural Networks	Applies AI (Graph Neural Networks) to classify auditory signals from multimodal data, pointing to automated assessment.
[30]	Muñoz Caracuel, M. (2024)	Systemic neurophysiological signals of auditory predictive coding	Investigates predictive coding; used in context of simultaneous EEG-fNIRS to correlate fast predictions with metabolic demands.
[31]	Jiang, C. (2024)	EEG and fNIRS Hybrid Brain Computer Interface Technology in Rehabilitation Medicine	Illustrates how combining EEG and fNIRS identifies robust biomarkers, applicable to auditory rehabilitation.
[32]	Ren, Y. et al. (2024)	A scoping review of utilization of the verbal fluency task in Chinese and Japanese clinical settings	Highlights standardization issues; reviewing Verbal Fluency Tasks reveals protocol heterogeneity mirroring audiology challenges.
[33]	Gallagher, A. et al. (2023)	fNIRS in pediatric clinical research: Different pathophysiologies and promising clinical applications	Validates fNIRS safety and utility in pediatric clinical research, reinforcing the call for standardized processing pipelines.
[34]	Zhang, F. et al. (2022)	Clenching-Related Motion Artifacts in fNIRS in the Auditory Cortex	Identifies jaw clenching artifacts mimicking auditory activation and proposes removal methods (Blind Source Separation).
[35]	Pinti, P. & Tachtsidis, I. (2023)	From Lab to Clinic: Overcoming Barriers to the Clinical Adoption of fNIRS	Critical analysis of barriers, specifically the high cost of equipment compared to EEG which hinders adoption.
[36]	Scholkmann, & Holper, L. (2022)	Integrating Advanced Neuroimaging into Clinical Practice: A Workflow and Cost-Benefit Analysis	Analyzes the lack of reimbursement models, noting fNIRS is often viewed as “experimental” by insurers.
[37]	Huppert, T.J. (2021)	Practical Considerations for fNIRS in Clinical Settings	Notes operational demands: while patient-friendly, data analysis is complex and requires specialized training.
[38]	Ross, J.M. et al. (2022)	A structured ICA-based process for removing auditory evoked potentials	Proposes automated ICA to clean data, representing future directions for easier clinical use.
[39]	Wang, Z. et al. (2023)	Rethinking Delayed Hemodynamic Responses for fNIRS Classification	Suggests rethinking classification of delayed hemodynamic responses to improve objectivity and diagnosis speed via ML.
[40]	Arif, A. et al. (2024)	EF-Net: Mental State Recognition by Analyzing Multimodal EEG-fNIRS via CNN	Introduces “EF-Net” (CNN) for mental state recognition, exemplifying AI use to automate multimodal data interpretation.
[41]	Adeli, B. et al. (2025)	AbsoluteNet: A Deep Learning Neural Network to Classify Cerebral Hemodynamic Responses	Presents a Deep Learning model to objectively classify whether a patient has “heard” a sound based on fNIRS data.

**Table 4:** Quantitative distribution of studies by the target population.

Target Population	Count	Interpretation of Trend	References
Adults	24	The dominance of adult studies reflects the phase of methodological validation and signal processing development.	[2,5,8,11,12,13,14,15,16,17,18,20,21,22,25,26,29,30,31,32,38,39,40,41]
Pediatrics	14	Although pediatrics is the target group, ethical and practical complexities lead to a lower absolute volume of publications.	[1,3,4,9,19,23,24,27,28,33,34,35,36,37]

**Table 5:** Distribution of studies by stimulation design.

Design Type	Count	Dominant Application	Reason for Preference in fNIRS	References
Block Design	28	Speech tests, listening in noise, continuous stimulation	Signal Robustness: Given the low signal-to-noise ratio (SNR) in fNIRS, block stimulation (e.g., 20 s stimulus/20 s rest) yields a stronger hemodynamic response than short events.	[1,5,8,9,13,15,16,17,19,20,21,22,23,24,25,28,29,31,32,33,34,35,36,37,38,39,40,41]
Event-Related	10	Threshold detection (tones), oddball paradigms (MMN)	Allows for the investigation of responses to single stimuli but requires longer inter-stimulus intervals (ISI) for hemodynamic recovery, prolonging testing time.	[2,3,4,11,12,14,18,26,27,30]

**Table 6:** Classification of studies by acoustic stimulus used.

Stimulus	Count	Clinical Insight	References
Speech	15	Highest Ecological Validity: Activates broad cortical networks (temporal and frontal lobes). Ideal for testing comprehension rather than just audibility.	[1,5,17,19,20,21,22,23,24,25,26,28,30,32,40]
Tones	8	Used for tonotopic mapping and threshold estimation. Less effective for eliciting robust hemodynamic responses than complex sounds.	[2,3,4,11,12,18,27,38]
Noise	6	Critical for testing “Listening Effort.” Noise serves as a stressor that reveals cognitive load.	[17,19,20,29,31,34]

**Table 7:** Comparison of studies by type of compensatory aid.

Device Type	Count	Reason for Disproportion	References
Cochlear Implant (CI)	9	Dominant: CIs produce massive electrical artifacts that obscure BERA/EEG signals. fNIRS is an optical method and is immune to electrical interference.	[20,21,22,26,27,28,29,31,33]
Hearing Aid	4	Less common, as traditional methods can often be used (e.g., removing the HA briefly or free-field BERA). fNIRS is used here primarily for assessing benefit in noise.	[5,12,17,19]

**Table 8:** Studies focusing on autism and developmental disorders.

Pathology	Count	Specific Findings	References
Autism/Dev. Disorders	4	Atypical speech lateralization, potential for guided stimulation.	[9,23,24,33]

## Data Availability

No new data were created or analyzed in this study. Data sharing is not applicable to this article.

## Abbreviations

The following abbreviations are used in this manuscript:

ABR	Auditory Brainstem Response
AEP	Auditory Evoked Potentials
BERA	Brainstem Evoked Response Audiometry
EEG	Electroencephalography
fMRI	Functional Magnetic Resonance Imaging
fNIRS	Functional Near-Infrared Spectroscopy
HbO	Oxyhemoglobin
HbR	Deoxyhemoglobin
ICA	Independent Component Analysis
MBLL	Modified Beer–Lambert Law
CI	Cochlear Implant
HA	Hearing Aid
