# Correction: Mimra et al. Functional Near-Infrared Spectroscopy (fNIRS) in Objective Audiometry: A Scoping Review and Clinical Perspectives. *Audiol. Res.* 2026, *16*, 3

**DOI:** 10.3390/audiolres16030085

**Published:** 2026-06-03

**Authors:** Tomáš Mimra, Martin Augustynek, Marek Penhaker, Lukáš Klein

**Affiliations:** 1 Department of Cybernetics and Biomedical Engineering, Faculty of Electrical Engineering and Computer Science, VSB—Technical University of Ostrava, 708 00 Ostrava, Czech Republic; 2 Department of Computer Science, Faculty of Electrical Engineering and Computer Science, VSB—Technical University of Ostrava, 708 00 Ostrava, Czech Republic; lukas.klein@vsb.cz; 3 ENET Centre—CEET, Faculty of Electrical Engineering and Computer Science, VSB—Technical University of Ostrava, 708 00 Ostrava, Czech Republic

Additional Affiliation

In the published publication [1], there was an error regarding the affiliation for Lukáš Klein. In addition to affiliation 3, the updated affiliation should include: Department of Computer Science, Faculty of Electrical Engineering and Computer Science, VSB—Technical University of Ostrava, 708 00 Ostrava, Czech Republic. The authors state that the scientific conclusions are unaffected. This correction was approved by the Academic Editor. The original publication has also been updated.
